# Vascular adventitial cells and vascular remodeling: when a harp of thousand strings does not keep in tune so long

**DOI:** 10.3389/fphys.2026.1866431

**Published:** 2026-08-24

**Authors:** Mani Sebastian, Anupama Vijayakumar, Parvathy Jayachandran, Mingyi Wang, Shivakumar Kailasam

**Affiliations:** 1 Department of Biotechnology, University of Kerala, Trivandrum, India; 2 Cardiovascular Genetics Laboratory, Department of Biotechnology, Bhupat and Jyothi Mehta School of Biosciences, Indian Institute of Technology Madras, Chennai, India; 3 Laboratory of Cardiovascular Science, National Institute on Aging/National Institutes of Health, Baltimore, MD, United States

**Keywords:** adventitia, aging, aneurysm, atherosclerosis, diabetes, ECM, hypertension, vascular adventitial fibroblasts

## Abstract

The tunica adventitia of blood vessels is a highly heterogeneous microenvironment comprising diverse cell types. Beyond providing structural scaffolding, adventitial fibroblasts act as dynamic, responsive cells that contribute to vascular homeostasis mainly through regulation of collagen, fibronectin and elastin expression and paracrine effects that promote vascular inflammation. Their dysregulation leads to the onset and progression of several diseases. Signaling pathways in adventitial fibroblasts, particularly DDR2, hypoxia-ERK1/2 MAPK and PI3K/Akt signaling, impact cell proliferation, pro-fibrotic gene expression and cellular response to stress. Furthermore, non-coding RNAs and novel epigenetic mechanisms significantly impact adventitial fibroblast function. Adventitial fibroblasts promote sex-related variations in the prevalence and severity of vascular diseases. Rapid advances in single-cell transcriptomics, biomarker development and other molecular and imaging techniques promise deeper insights into adventitial fibroblast biology, unravelling their heterogeneity and functional variability, and their role in extracellular matrix remodeling and paracrine signaling. These in turn would pave the way for innovative strategies to target pro-fibrotic fibroblast subpopulations and prevent adverse vascular remodeling. This review provides an overview of adventitial fibroblast function and the role of these cells in atherosclerosis, hypertension, diabetes, aneurysm and age-related vascular dysfunction, underscoring the need for a paradigm shift in vascular biology, positioning the adventitia as a critical regulator of vascular health and disease.

## 1 Introduction

One of the earliest recognized functional anatomical systems, the vasculature is a vital circulatory network of arteries, veins and capillaries involved in the transport of gases, nutrients and metabolic waste products, and in regulating blood pressure and maintaining vascular tone (Chetty et al., 2017; Iruela-Arispe and Zovein, 2017; Li et al., 2024; Paul et al., 2021; Slowey and Nyhan, 2022; Von Nieda, 2007). Vascular cells, comprising endothelial cells, vascular smooth muscle cells (VSMCs) and fibroblasts, are genetically programmed to respond to physiological and pathophysiological stimuli such that blood flow is maintained to cope with altering metabolic demands of the tissue (Rudic and Sessa, 1999).

Vascular remodeling is a complex and active process of dynamic changes in the endothelium, fibroblasts, VSMCs and pericytes that impact cell growth, cell death, cell migration and refurbishing of the extracellular matrix (ECM) scaffold, leading to adaptive structural changes (Renna et al., 2013; Totoń-Żurańska et al., 2024; Zeng and Yang, 2024; Gibbons and Dzau, 1994; Lyle and Taylor, 2019; Schiffrin, 2012). Although vascular remodeling begins as an adaptive process, it can become maladaptive in response to long-term hemodynamic alterations, leading to impaired vascular function eventually (Bagmet, 2002). Pathological vascular remodeling associated with vascular dysfunction marks diverse cardiovascular pathologies, including hypertension, diabetes, heart failure, atherosclerosis and vascular aneurysm (Lee et al., 2019; Stenmark et al., 2011; Totoń-Żurańska et al., 2024). While endothelial cells of the intima have sophisticated sensory and regulatory ability to maintain vascular homeostasis through adaptive remodeling, VSMCs of the medial layer are a major driver of vascular remodeling. However, the principal “injury-sensing tissue” of the vessel wall is the adventitial compartment. Adventitial fibroblast is the primary responder and initiator of vascular remodeling (Baker et al., 2008; Gibbons and Dzau, 1994; Stenmark et al., 2011). Different types of fibroblast clusters, each with a unique set of differentially-expressed genes, are found in both healthy and diseased tissues. Research on regional specificity of fibroblasts has demonstrated that new fibroblast clusters emerge in pathological conditions, which highlights the plasticity and heterogeneity of these cells (Xie et al., 2018).

Since adventitial fibroblasts are a major determinant of ECM turnover in the vessel wall, this review focuses on their role in atherosclerosis, hypertension, diabetes, vascular aneurysm and the normal process of vascular aging that are marked by significant changes in ECM, leading to compromised structural and functional integrity of the vasculature. The review also examines the underlying mechanisms and provides a snapshot of potential avenues for future research, including the identification of novel therapeutic strategies targeting adventitial fibroblasts.

## 2 Materials and methods

### 2.1 Search strategy

A comprehensive literature search was conducted to identify studies investigating the role of adventitial fibroblasts in vascular pathophysiology, including atherosclerosis, hypertension, diabetes, aneurysm formation, and age-related vascular dysfunction. Electronic databases such as PubMed, Scopus, Google Scholar and Web of Science were searched for relevant articles published up to 2026. The search strategy employed combinations of keywords and Boolean operators (AND/OR), including “adventitial fibroblasts,” “vascular adventitia,” “atherosclerosis,” “hypertension,” “diabetes,” “vascular remodeling,” “aneurysm,” “vascular aging,” “fibrosis,” “extracellular matrix,” “DDR signaling,” and “inflammation.”

### 2.2 Eligibility criteria

All retrieved data were imported into Mendeley reference management software, and duplicate entries were removed prior to screening. Study selection was performed in two stages, an initial screening of titles and abstracts to assess relevance, followed by full-text evaluation of potentially eligible articles based on predefined inclusion and exclusion criteria. Studies were included if they comprised original research articles, reviews, or experimental studies focusing on adventitial fibroblasts and their involvement in vascular diseases, including *in vitro*, *in vivo*, or clinical investigations published in English. Studies were excluded if they did not specifically address adventitial fibroblasts, were non-peer-reviewed publications such as editorials or conference abstracts without full text or lacked sufficient methodological detail.

### 2.3 Data extraction

Data extraction was performed using a standardized approach to collect relevant information from each included study, including study characteristics, experimental models, source and characterization of adventitial fibroblasts, disease context, and key molecular mechanisms. Particular emphasis was placed on signaling pathways such as TGF-β, DDRs, oxidative stress pathways, and inflammatory mediators. The primary outcome measures assessed included markers of AdF activation (such as α-smooth muscle actin expression and fibroblast-to-myofibroblast transition), extracellular matrix remodeling (including collagen deposition and fibrosis), inflammatory responses (cytokine and chemokine production), oxidative stress indicators, and the involvement of key signaling pathways. Additionally, the functional contribution of adventitial fibroblasts to vascular remodeling, stiffness and disease progression was evaluated.

## 3 Vascular adventitial fibroblasts

**Figure 1:**
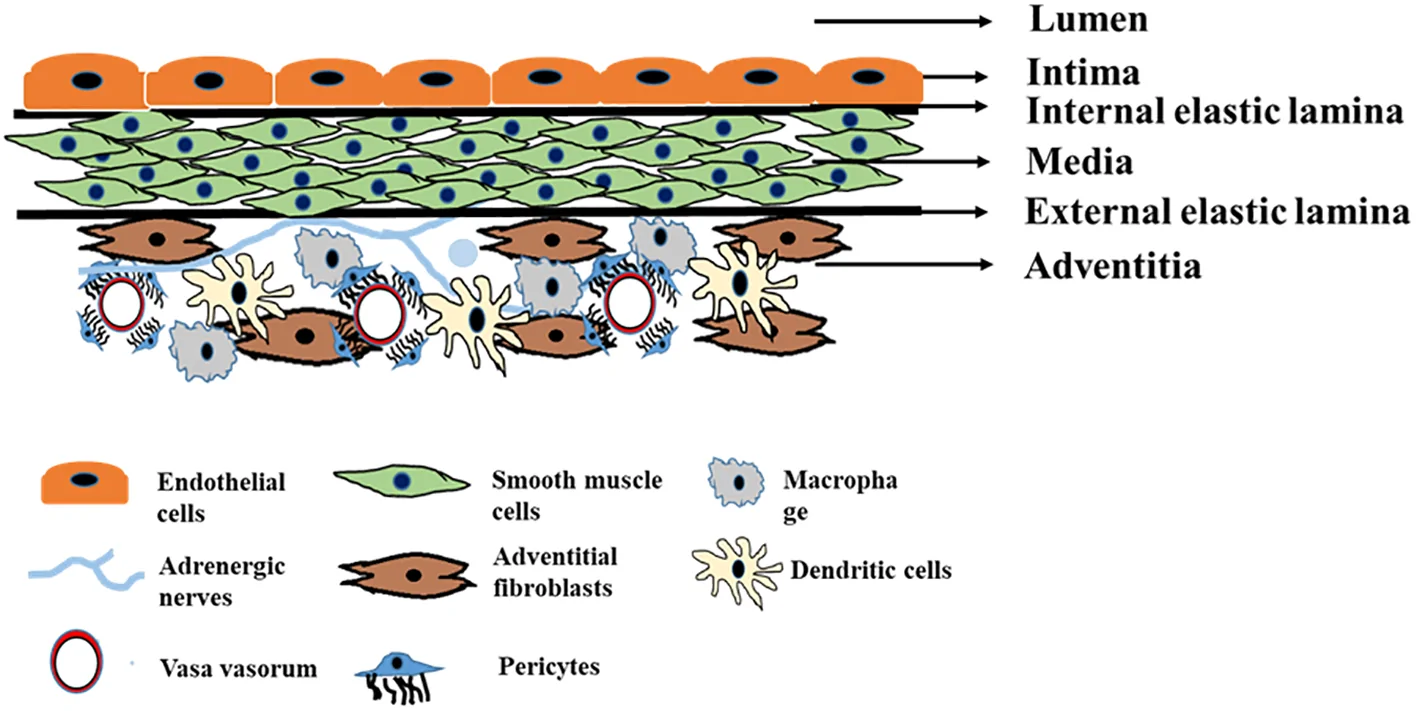
Arrangement of different cell types in the different layers of the vascular wall.

**Figure 2:**
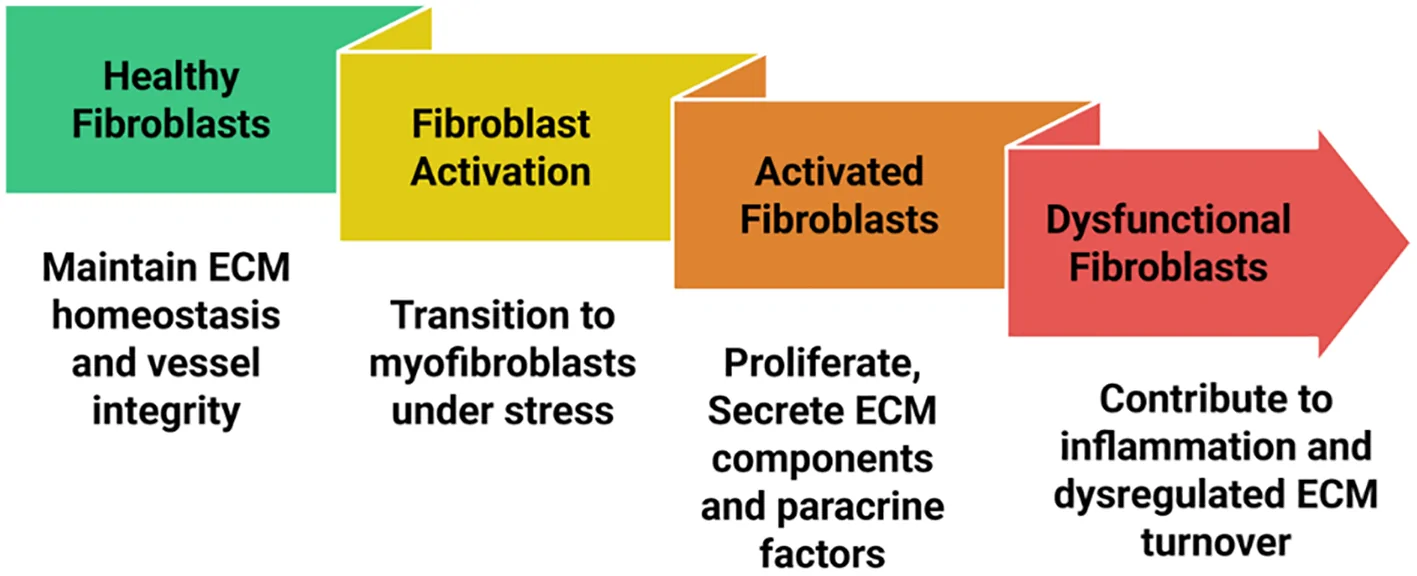
Role of adventitial fibroblasts in vascular remodeling.

The vessel wall is a three-layered structure comprising the intima, media and adventitia (**Figure 1**) (Laffan and Manning, 2006), each exhibiting specific histological, biochemical and functional behavior and contributing to vascular homeostasis and vascular response to stress and injury (Stenmark et al., 2011). The innermost layer of the blood vessel, the intima, consists of a monolayer of endothelial cells that form a smooth and non-adhesive surface to facilitate blood flow and prevent clotting (Dudás, 2023). These cells act as a sensor of blood flow, regulate vascular processes, communicate with other cell types like macrophages, fibroblasts and VSMCs, and mediate leukocyte adhesion and inflammation. Media is the middle layer of the blood vessel, located between the intimal and adventitial layers. VSMCs, the predominant medial cell type embedded in a matrix of elastin fibers and collagen, regulate vessel contractility and stability, controlling blood flow and blood pressure. They play a critical role in atherosclerosis through migration to the intimal layer and proliferation (Brozovich et al., 2016). The outer adventitia comprises adventitial fibroblasts, lymphatic vessels, immune cells and nerves that make the layer complex and sensitive to external stimuli (H. Di Wang et al., 2010). The adventitial layer, with an abundance of collagen and elastin arranged as alternating layers (Haas et al., 1991), plays a pivotal role in the response to injury, inflammation, hypoxia and mechanical stress through communication with cells within the adventitia and neighboring tissues (An et al., 2015; Haas et al., 1991; Haurani and Pagano, 2007; Majesky, 2015; Tinajero and Gotlieb, 2020). A relatively understudied cell type, the vascular adventitial fibroblast has recently drawn attention as a dynamic player in vascular pathophysiology (**Figure 2**) (Majesky, 2015; Michel et al., 2007). As a major source of collagen, elastin and fibronectin, adventitial fibroblasts help maintain vascular structure and function by providing structural support to blood vessels and maintaining their elasticity and strength (Liang et al., 2018; Michel et al., 2007; Xu and Shi, 2014).

The collagen in the adventitial layer helps the adventitia withstand the tension due to blood pressure (Holzapfel et al., 2000; Stenmark et al., 2013), enables the fibroblasts to crawl over the layer and migrate (Dallon and Sherratt, 1998) and provides a scaffold for other cell types to adhere and grow (Reinke and Sorg, 2012). Further, elastin and fibronectin, produced by adventitial fibroblasts, help maintain the elasticity and resilience of blood vessels and ensure the structural integrity and mechanical properties of the vessel wall (Hedman et al., 1978; Hynes and Yamada, 1982).

In response to injury or stress, adventitial fibroblasts undergo transformation into activated α-smooth muscle actin (α-SMA)- or desmin-expressing myofibroblasts that show increased contractile activity, migration, proliferation, increased DNA content, elevated cytokine and chemokine production, release of growth factors, activation of signaling pathways, vascular hypertrophy, enhanced ECM remodeling and expression of surface adhesion molecules such as VCAM-1 that recruit inflammatory mediators from the perivascular tissue (Chatelain and Dardik, 1988; Coen et al., 2011; Schickling and Miller, 2021; Schmitt-Gräff et al., 1994; Scott et al., 2020; Tinajero and Gotlieb, 2020). All of these facilitate wound healing and remodeling in response to vascular injury (Shi et al., 1997; Strauss and Rabinovitch, 2000; Xu et al., 2022). Dysfunctional fibroblasts promote arterial stiffness in conditions like pulmonary arterial hypertension, compromising vessel compliance and contributing to vascular pathologies (Lehoux, 2016; Niestrawska et al., 2019; Kuwabara and Tallquist, 2017; Tinajero and Gotlieb, 2020; Wang et al., 2010). Recent studies have highlighted the concept of “outside-in” signaling, wherein vascular adventitial fibroblasts sense and respond to external stimuli from the surrounding tissue layers, triggering global changes in the vessel wall. This concept underscores the bidirectional communication between the adventitia, media and intima.

## 4 Heterogeneity of adventitial fibroblasts

Recent advances in single‐cell transcriptomics and genetic lineage tracing show distinct subpopulations of adventitial fibroblasts, each characterized by a unique set of gene expression profile and functional properties (Bashore et al., 2025; Fries et al., 2025). Corselli et al. (2012) expanded the perivascular origin model of mesenchymal stem cells (MSCs) by identifying a distinct progenitor population residing within the tunica adventitia of large human blood vessels. Sorted from human white adipose tissue as CD34^+^,CD31^-^,CD146^-^,CD45^-^, these native adventitial cells gave rise to multipotent, clonogenic progenitors identical to standard bone marrow-derived MSCs in culture. While adventitial cells and microvascular pericytes maintain distinct phenotypes and genotypes under standard conditions, they coexist as two separate perivascular MSC sources. Furthermore, the study demonstrated unique cellular plasticity, showing that adventitial cells can transition to a pericyte-like phenotype when exposed to growth factors involved in vascular remodeling (Corselli et al., 2012). By using single-cell RNA sequencing and spatial transcriptomics on human arteries and veins, Wang et al. (2024) uncovered a highly stratified cellular architecture within the tunica adventitia. The study identified CD201 as a highly specific marker that maps exclusively to the outermost boundary of this adventitial layer. This distinct CD34^+^, CD201^+^ subpopulation represents a primitive, early-stage mesenchymal progenitor pool that exhibits superior clonogenic and proliferative capacity compared to standard perivascular cells. Functionally, these progenitors are maintained in a quiescent, undifferentiated state within their specialized spatial niche by localized active signaling pathways such as BMP signaling (Wang et al., 2024). Studies on genetic fate-tracing in mice identified Gli1^+^ as a marker for a distinct subpopulation of resident adventitial mesenchymal stem cell-like progenitors (Kramann et al., 2016). Under physiological conditions, these adventitial Gli1^+^ cells contribute to vascular homeostasis by serving as a baseline source of new vascular smooth muscle cells. However, under pathological conditions such as chronic kidney disease (CKD), these progenitors undergo a detrimental lineage fate switch. In response to chronic uremic stress, the Gli1^+^ cells proliferate, migrate from the adventitia into the media and neointima, and differentiate into bone-forming osteoblast-like cells. The study demonstrates that this specific adventitial progenitor subset is a major cellular driver of arterial remodeling, fibrosis, and vascular calcification in kidney disease, making them a prime therapeutic target. When vessels are subjected to different contexts like hypertension or hypoxia, a subset of fibroblasts gets activated, promoting increased proliferation, migration, and secretion of ECM proteins and pro-fibrotic cytokines (Crnkovic et al., 2024; Solinc et al., 2022). scRNA-seq analyses of the aortic adventitia in LDLR^−/−^ mice identify four distinct fibroblast populations that undergo dynamic transcriptomic shifts during atherogenesis. During disease progression, adventitial fibroblasts transition from homeostatic states to activated, migratory phenotypes. *In vitro* validation confirms that SERPINH1 knockdown impairs fibroblast migration and alters subcluster-specific gene expression. These findings position the adventitia as a primary hub, where GWAS-linked genes like SERPINH1 drive cellular heterogeneity and maladaptive remodeling that facilitate atherosclerosis (Fries et al., 2025). Yet another scRNA-seq mapping of the murine and human adventitia established PDGFRA and DPEP1 as robust, specific fibroblast markers, distinguishing them from less specific markers like CD90. Functional heterogeneity is defined by three different differentiation trajectories CD55+, CXCL14+, and LOX+ that correlate with vascular development, antigen presentation, and collagen organization, respectively. These clusters expand in response to aging and hypercholesterolemia, driving adventitial fibrosis. The enrichment of CVD-linked GWAS genes within these trajectories and their correlation with human plaque traits underscore the translational importance of fibroblast diversity in directing lipid-independent vascular remodeling (van Kuijk et al., 2023). Lineage tracing has shed light on how populations of adventitial fibroblasts or their progenitors contribute to neointimal formation and vascular adventitial stiffening post-injury (Shi et al., 1996). Adventitial fibroblasts may originate from local mesenchymal progenitors or transdifferentiate from other resident cell types or even arise from epithelial-to-mesenchymal transition (EMT) and endothelial-to-mesenchymal transition (EndMT) during disease progression. These divergent origins may lead to differences in proliferative capacity, secretory profiles, interactions with neighboring cells and contributions to vascular pathology (Gittenberger-de Groot et al., 1998; Lendahl et al., 2022; Plikus et al., 2021).

## 5 Adventitial fibroblasts in vascular disease

Dysregulation of matrix turnover in adventitial fibroblasts marks conditions like atherosclerosis, hypertension, diabetes, vascular aneurysm and aging, as would be evident from the following overview (**Tables 1**, **2**).

**Table 1:** Preclinical animal models utilized in studies on adventitial biology and vascular disease.

Experimental model	Pathology	Impact on adventitial layer	Reference
Apolipoprotein E-Deficient Mouse (ApoE^-^/^-^)	Chronic Atherosclerosis & “Outside-In” Vascular Inflammation	Triggers early adventitial expansion, robust macrophage/T-cell infiltration, tertiary lymphoid organogenesis, and differentiation of adventitial stem cells into pro-fibrotic myofibroblasts.	(Dubner et al., 2023)
Hyperlipidemic Swine/Porcine Model)	Plaque Complexity & Vascular Remodeling	Induces massive adventitial vasa vasorum neovascularization and angiogenesis, directly driving positive geometric vessel remodeling, smooth muscle cell depletion, and advanced intraplaque macrophage infiltration.	(Alviar et al., 2010)
Genetically Tracked Gli1^+^ Mouse	Chronic Kidney Disease (CKD)-Associated Arterial Calcification	Identifies a distinct Gli1^+^ resident adventitial progenitor pool that proliferates and undergoes an osteogenic lineage switch, migrating into the media/neointima to drive pathological medial calcification.	(Kramann et al., 2016)
Hutchinson-Gilford Progeria (HGPS) Mouse(Transgenic LMNA G608G point mutation or progerin overexpression)	Accelerated Vascular Aging & Premature Fibrosis	Recapitulates severe vascular aging, inducing profound adventitial fibrosis, massive extracellular matrix (ECM) disorganization, adventitial fibroblast senescent phenotypes, and vessel stiffening.	(Benedicto et al., 2021)
Angiotensin II Infusion Rodent Model	Hypertension-Induced Aortic Remodeling & Aneurysm Formation	Promotes rapid adventitial fibroblast proliferation, excessive collagen deposition, upregulation of reactive oxygen species (ROS), and infiltration of circulating leukocytes via the adventitial layer.	(Kuwabara and Tallquist, 2017)
Post-Mortem Vascular Autopsies	Age-Dependent Arterial Stiffening & Senescence	Natural vascular changes after death	(Egger et al., 2023)

**Table 2:** Adventitial fibroblasts in vascular pathology.

Atherosclerosis • Phenotypic switch to myofibroblasts; migration to the intima to form the fibrous cap (Shi et al., 1996; Siow et al., 2003) • Pro-inflammatory cytokine release (IL-6, MCP-1) (Wang et al., 2012) • Contribution to plaque stability and neointima formation (Dubner et al., 2023)
Aneurysm • Excessive Proteolysis, degradation of the structural scaffold and elastic lamina • MMP activation (MMP-2, MMP-9) (Lin and Davis, 2023)
Hypertension • Hyperplasia and deposition of stiff, disorganized collagen (Zhang et al., 2024) • Responsible for mechanical stretch and TGF-β/Smad signaling • Focus on medial thickening and increased arterial stiffness
Diabetes (Remodeling) • Metabolic reprogramming and glycation-induced ECM crosslinking • Chronic inflammation, TGF-β pathway and oxidative stress (NADPH oxidase) • Formation of AGEs (Advanced Glycation End-products) that “lock” the vessel in a stiff state (Liu et al., 2010; Zhang et al., 2003)
Aging • Cellular senescence and the Senescence-Associated Secretory Phenotype (SASP) (Sarad et al., 2024) • Increased ROS, reduced quality of collagen turnover and chronic inflammation • Loss of regenerative capacity, shift from organized collagen to disordered, calcified fibers

### 5.1 Atherosclerosis

A chronic inflammatory disease of the arterial wall, atherosclerosis is characterized by accumulation of lipids, inflammatory cells and fibrous tissue within the intimal layer of large and medium-sized arteries. The conventional “inside-out” theory of atherosclerosis postulates that the inflammatory response progresses from the intimal layer to the adventitial layer, thus stressing the involvement of endothelial dysfunction, lipid deposition, inflammation, and proliferation and migration of VSMCs.

#### 5.1.1 The “outside-in” hypothesis

A more recent and contrarian view, however, is the “outside-in” hypothesis that the initial trigger for atherosclerotic lesion formation and inflammation comes from the adventitial layer and progresses to the intimal layer. In response to pathogenic stimuli, fibroblasts originating from the adventitia or circulating fibrocytes undergo activation and promote intimal atherosclerotic lesion formation (Badimon et al., 2012; Schickling and Miller, 2021; Scott et al., 1996; Xu et al., 2007a). Activated fibroblasts promote ECM remodeling, formation of a fibrous cap and stabilization of the atherosclerotic plaque (Halper, 2018). Moreover, fibroblasts secrete MMPs and TIMPs that regulate ECM turnover, influencing plaque vulnerability. The adventitial layer serves as an active compartment for inflammatory cells. Moreover, ECM proteins permeate the adventitial layer and facilitate interactions between cells inside and outside the vessel. Historically, adventitia was viewed as a passive, supportive connective tissue. However, over three decades of vascular research has shown that they may not always initiate the disease but may play a critical, active role in plaque progression, arterial remodeling and plaque rupture. The traditional view adheres to the classic “Inside-Out” theory which places the initiation of atherosclerosis at the luminal surface through endothelial dysfunction and lipid accumulation. However, robust evidence supports a compelling contrarian paradigm known as the “Outside-In” hypothesis (Maiellaro and Taylor, 2007). This model posits that vascular inflammation and subsequent atherogenesis can be actively driven by signals originating in the outermost layer of the vessel wall, propagating inward toward the media and intima. The major hallmarks of the outside-in cascade involve a highly coordinated interplay between resident adventitial cells, infiltrating immune populations, and structural microvessels. According to Maiellaro and Taylor (2007), under vascular stress such as mechanical overdistension, metabolic imbalance, hypercholesterolemia, or hypoxia, the adventitia may act as a sensor of injury in the vessel wall (Maiellaro and Taylor, 2007; Sakamoto et al., 2014; Stenmark et al., 2013). Upon activation, the resident cell population undergoes phenotypic reprogramming. Exogenous immune cells, including monocytes, macrophages, and lymphocytes, progressively populate the adventitial space. Simultaneously, resident adventitial fibroblasts undergo a key phenotypic switch into highly migratory, proliferative myofibroblasts (Kuwabara and Tallquist, 2017). These activated fibroblasts upregulate cell adhesion molecules, produce excess extracellular matrix proteins, alter the local redox state through the generation of reactive oxygen species (ROS), and secrete a potent cocktail of inflammatory chemokines and cytokines. The localized, self-sustaining inflammatory microenvironment generates an inward-propagating wave of cellular activation that directly destabilizes the underlying medial smooth muscle cells and luminal endothelium. As reviewed by Kawabe and Hasebe (2014), changes in the density and structural integrity of the vasa vasorum are closely associated with, and frequently precede, the development of advanced intimal atheromatous plaques. In a healthy vessel, the vasa vasorum serves its conventional homeostatic function as a blood conduit tube, delivering vital oxygen and nutrients to the outer layers of large arterial walls. Under hypercholesterolemic or inflammatory stress, however, the vasa vasorum undergoes angiogenesis and neovascularization. These fragile, leaky neovessels act as a major cellular conduit, facilitating the accelerated delivery of circulating leukocytes, pro-inflammatory cytokines, and oxidized lipids into the deep vessel wall (Kawabe and Hasebe, 2014).

#### 5.1.2 Role of adventitial fibroblasts in atherosclerosis

##### 5.1.2.1 Dysregulated ECM turnover

In response to inflammatory stimuli and mechanical stress, activated αSMA^+^ adventitial fibroblasts migrate to the intima where they trigger an increase in collagen synthesis, kick-starting the initial phase of plaque development (Tinajero and Gotlieb, 2020), which indicates a role for these cells in the formation of neointima (Dubner et al., 2023; Shi et al., 1996; Siow et al., 2003). The most expressed collagen types in the adventitial layer are I, III, IV, V, and VI although expression array data suggest the presence of 17 distinct forms of collagen in mouse aorta (Kelleher et al., 2004). While collagen in the fibrous cap is traditionally attributed to resident plaque fibroblasts, it has recently been suggested that a significant contribution is from adventitial fibroblasts that have migrated through the media (Xu et al., 2007b). This migration is driven by a phenotypic switch to a myofibroblast state, triggered by adventitial-derived ROS and Shh-signaling (Passman et al., 2008). Furthermore, the distinction between these populations is increasingly blurred as both VSMC-derived ‘fibromyocytes’ and adventitial-derived myofibroblasts contribute to the ECM architecture of the plaque (Wirka et al., 2019).

##### 5.1.2.2 Fibronectin and elastin

Fibronectin plays a role in cell adhesion, migration and matrix assembly and promotes the progression of atherosclerosis through ECM remodeling and plaque stability (Gauthier et al., 2023). Overexpression of fibronectin type III domain-containing 5 (FNDC5) reduces reactive oxygen species production in adventitial fibroblasts from Spontaneously Hypertensive Rats and hinders phenotypic alterations within adventitial fibroblasts, showing that FNDC5 may ameliorate vascular inflammation, hypertension and remodeling in the SHR model (Ling et al., 2018). Fibroblasts also contribute to elastin synthesis and remodeling in response to mechanical and biochemical cues. Changes in the structure and function of elastin, which provides elasticity to blood vessels and resilience to tissues (Cocciolone et al., 2018), also contribute significantly to atherosclerosis progression. III. MMP/TIMP imbalance.

Activated fibroblasts produce MMPs and inhibitors of MMPs that determine collagen turnover, thereby impacting plaque stability and clinical outcomes in atherosclerosis (Singh and Torzewski, 2019). MMPs, particularly MMP-1, MMP-8 and MMP-13, are key enzymes involved in collagen degradation in LDLR^-/-^Apob100/100 mouse model (Dick et al., 2011) and are secreted by various cell types within the plaque, including macrophages, smooth muscle cells and fibroblasts. TIMPs control MMP activity and regulate ECM turnover, tissue remodeling and cellular behavior (Cabral-Pacheco et al., 2020). Fibroblast-like cells originating from EndMT contribute significantly to plaque destabilization. Phenotypic cell switching through EndMT can potentially drive atherosclerosis progression by modifying the balance between collagen and MMP. The presence of EndMT-derived fibroblasts in complex lesions and ruptured plaques in humans underscores the clinical significance of this process (Evrard et al., 2016). Periostin, a matricellular protein that is highly expressed in blood vessels, modulates the expression and activity of MMPs and TIMPs and influences ECM turnover and adventitial remodeling in atherosclerosis (Wang et al., 2022). Elevated expression of periostin activates adventitial fibroblasts and contributes to adventitial remodeling in ApoE-/- mice model (Wang et al., 2022). In a mouse model of atherosclerosis, loss of periostin is reported to delay disease progression by altering mature collagen production and modifying the migratory potential of macrophages (Schwanekamp et al., 2016), pointing to periostin as a potential drug target to slow the progression of atherosclerosis.

#### 5.1.3 Paracrine effects of adventitial fibroblasts in atherosclerosis

Adventitial fibroblasts exert paracrine effects on different cell types involved in atherosclerosis, which indicates the complex cellular and molecular processes underlying disease pathogenesis.

##### 5.1.3.1 Endothelial cells

Fibroblasts regulate endothelial cell function (Enzerink and Vaheri, 2011). Adventitial fibroblasts express vascular endothelial growth factor receptor 1 (VEGFR1) and also produce VEGF (Jin et al., 2007) that augments vasa vasorum count, aiding macrophage recruitment and neointima formation (Li et al., 2020).

##### 5.1.3.2 Smooth muscle cells

Fibroblast-derived factors such as TGF-β and PDGF stimulate VSMC proliferation and migration to the intima, contributing to intimal hyperplasia and plaque formation. Additionally, fibroblast-secreted ECM proteins may modulate VSMC adhesion, migration and contractility within the arterial wall. Conditioned media from calcified adventitial fibroblasts elicit differential calcification responses in VSMCs, suggesting that adventitial fibroblasts might mediate VSMC calcification (Kennon and Stewart, 2023). Stimulation of fibroblasts with Shh and PDGF-BB induces the secretion of IL-6, IL-8, and CXCL1, which drive inflammatory responses, SMC proliferation and migration (Chava et al., 2009; Shoji et al., 2014; Yue et al., 1994).

##### 5.1.3.3 Macrophages

Fibroblasts recruit macrophages to the inflamed sites that in turn enhance fibroblast immunoactivation. Fibroblasts co-cultured with macrophages induce contact-dependent expression of chemokine (C-C motif) ligand 3) CCL3 that amplifies inflammatory signals (Lukacs et al., 1994). Additionally, adventitial fibroblast-generated ROS and MCP-1 can attract monocytes (Liu et al., 2003; Tieu et al., 2011) that promote fibroblast proliferation, adventitial thickening and cytokine secretion. This loop involving fibroblasts and monocytes can impact adventitial inflammation associated with various cardiovascular diseases (Tieu et al., 2011).

#### 5.1.4 Signaling pathways in adventitial fibroblasts implicated in atherosclerosis

It has been reported that lipopolysaccharide activates TLR4 in human adventitial fibroblasts, leading to NF-κB-mediated secretion of IL-6, MCP-1 and TNF-α, and increased lipid uptake, which links innate immune signaling to metabolic changes in atherosclerosis (Wang et al., 2012). Vink et al., 2002 demonstrated that Toll-Like Receptor 4 (TLR4) is expressed in both human atherosclerotic plaques and adventitial cells, including macrophages and primary adventitial fibroblasts. *In vitro* studies showed that stimulating these isolated human adventitial fibroblasts with lipopolysaccharide (LPS) activates the NF-κB pathway and upregulates various pro-inflammatory cytokines and chemokines. To examine this mechanism *in vivo*, researchers utilized a mouse femoral artery cuff model and discovered that peri-adventitial application of LPS significantly augmented neointima formation. On the other hand, when the same procedure was performed on TLR4-defective mice, the resulting neointimal lesion was significantly smaller than that observed in wild-type mice. These findings provide direct evidence connecting adventitial TLR4 activation to intimal lesion development (Vink et al., 2002).

Ling et al. (2017) and Wang et al. (2022) showed that periostin is significantly upregulated in the adventitia of atherosclerotic aortae in ApoE^-^/^-^ mice, which promotes adventitial fibroblast activation via integrin-mediated phosphorylation of FAK and Src, resulting in increased expression of α-SMA, TGF-β1, MCP-1, COL1A1, COL3A1, and MMP-2/9. The study also highlighted a positive feedback loop where TGF-β1 further induces periostin expression, causing ECM remodeling (Ling et al., 2017; Wang et al., 2022). Studies on rat models of balloon-induced arterial injury showed that administration of ERK1/2 MAPK inhibitors to the adventitial surface significantly reduced neointimal formation and lowered the intima-to-media ratio (Chen et al., 2024). Inhibition of ERK1/2 MAPK also prevented PDGF-BB-induced G1 phase release and led to downregulation of Cyclin D1 and GATA4, key regulators of cell cycle progression and gene expression (Chen et al., 2024). In addition, Angiotensin II-induced migration of adventitial fibroblasts in Spontaneously Hypertensive Rats (SHR) was mediated via ERK1/2 MAPK activation. This migratory response was blocked by the ERK1/2 inhibitor, PD98059, and the AT1 receptor antagonist, losartan, suggesting the involvement of Angiotensin II signaling through this MAPK pathway. These findings establish ERK1/2 MAPK as a central effector in the biochemical and biomechanical regulation of adventitial fibroblasts (Li et al., 2006).

### 5.2 Adventitial fibroblasts in hypertension-induced vascular remodeling

Adventitial fibroblasts are a key cellular contributor to vascular remodeling in hypertension. In models such as SHR, adventitial fibroblasts show altered phenotypic behavior, including increased proliferation, migration and cytokine production, thereby driving structural and functional changes in the vessel wall (Li et al., 2006; Wang et al., 2025; Zhu et al., 1991). They interact with endothelial cells, VSMCs and infiltrating immune cells, creating a microenvironment that supports inflammation and fibrosis. Prasad et al., 2025 showed that the intracellular complement system acts as a primary driver of pathological changes in vascular fibroblasts during pulmonary hypertension. By evaluating human tissue and bovine models, the researchers discovered a significant upregulation and heightened activation of key intracellular complement proteins, specifically C3, CFB, and CFD, within pulmonary hypertension fibroblasts. This intracellular complement surge triggers profound metabolic reprogramming and elevates the secretion of pro-inflammatory mediators like MCP1, IL-6, and IL-33. Remarkably, silencing CFD via shRNA reversed these effects by suppressing C3a production and normalizing essential metabolic pathways, including glycolysis, fatty acid metabolism, and tricarboxylic acid (TCA) cycle activity (Prasad et al., 2025).

#### 5.2.1 Signaling pathways in adventitial fibroblasts implicated in hypertension

A hallmark of hypertensive signaling in adventitial fibroblasts is Angiotensin II-induced oxidative stress. Harrison et al. (2021) demonstrated that fibroblast-specific Nox2 activation promotes ROS generation, which in turn stimulates paracrine signaling to vascular smooth muscle cells, leading to medial hypertrophy and fibrosis (Harrison et al., 2021). The redox-sensitive regulation of gene expression and matrix production places NOX enzymes at the center of adventitial fibroblast-driven vascular remodeling. Transforming Growth Factor-β (TGF-β) signaling, a well-known profibrotic pathway, is upregulated in hypertension. On activation, TGF-β binds to its receptors (TGF-βR I/II), triggering phosphorylation of SMAD2/3, which form complexes with SMAD4 and translocate to the nucleus. In adventitial fibroblasts, this pathway drives collagen synthesis, α-SMA expression and myofibroblast differentiation, making it a pivotal mechanism contributing to ECM accumulation in the hypertensive vessel and consequent stiffening (Renna et al., 2013; Zeng and Yang, 2024). MAPK signaling, including the ERK1/2 MAPK cascade, is activated in fibroblasts by mechanical stress, Ang II and PDGF. In hypertensive models, ERK1/2 MAPK promotes adventitial fibroblast proliferation and migration (Schiffrin, 2012). It also synergizes with TGF-β signaling to enhance pro-fibrotic gene expression, amplifying the hypertrophic remodeling response. Further, the JAK/STAT pathway mediates inflammatory cytokine signaling in adventitial fibroblasts. Activation by IL-6 leads to STAT3 nuclear translocation, inducing transcription of proliferative and inflammatory genes. Zeng and Yang (2024) describe this pathway as an important link between chronic inflammation and fibrosis in hypertensive vessels, with adventitial fibroblasts playing a central role in perpetuating this feedback loop (Zeng and Yang, 2024). Furthermore, Ling et al. (2018) have shown that the NLRP3 inflammasome and NF-κB signaling contribute to a proinflammatory phenotype in adventitial fibroblasts from SHR, which leads to enhanced secretion of IL-1β, IL-6 and TNF-α, supporting leukocyte recruitment and further remodeling of the vessel wall (Ling et al., 2018). PDGF-BB binding to PDGFRα/β receptors on adventitial fibroblasts triggers PI3K-Akt and MAPK pathways, promoting cell proliferation, migration and ECM remodeling. Solinc et al. (2022) demonstrated that PDGFRα activation in progenitor fibroblast-like cells is a major driver of vascular remodeling in pulmonary hypertension, with likely parallels in systemic hypertension (Solinc et al., 2022). The Notch1–Jagged1 axis modulates fibroblast activation, differentiation and cross-talk with endothelial and immune cells. Activation of Notch in adventitial fibroblasts leads to increased α-SMA expression and matrix deposition. This is particularly relevant in pressure-overload models where Notch cooperates with TGF-β to sustain myofibroblast transition. Though less studied in hypertensive adventitia, emerging evidence suggests that it may be a key regulator of fibroblast fate (Zeng and Yang, 2024). Noncoding RNAs, particularly miR-29a-3p, suppress fibrosis-associated genes such as COL1A1 and α-SMA in adventitial fibroblasts (Luo et al., 2015). Wu et al. (2018) review multiple ncRNAs that regulate hypertensive remodeling through control of fibroblast proliferation and ECM gene expression (Wu et al., 2018). Extracellular vesicles containing miRNAs represent a novel mechanism of cell-cell communication in the hypertensive vasculature (Zhang and Sun, 2022). In aging-related hypertension, adventitial fibroblasts may adopt a senescent or proinflammatory phenotype (Sun, 2015), contributing to arterial stiffness and altered vascular compliance. Thus, age-related changes in fibroblast function should be integrated into future models of hypertensive remodeling.

### 5.3 Adventitial fibroblasts in diabetes-induced vascular remodeling

In hyperglycemia, normally quiescent adventitial fibroblasts get activated, differentiate into myofibroblasts, migrate, proliferate and secrete extracellular matrix proteins and matrix metalloproteinases, which leads to adventitial thickening and fibrosis (Kuwabara and Tallquist, 2017; Wang et al., 2020). Diabetes-related stimuli induce a pro-inflammatory phenotype while high glucose and advanced glycation end products (AGEs) upregulate expression of cytokines (IL-6, TNFα), chemokines (MCP-1/CCL2) and adhesion molecules (VCAM-1) in adventitial fibroblasts (Liu et al., 2010; Zhang et al., 2003). In diabetic vessels, increased reactive oxygen species production by adventitial fibroblasts further amplifies inflammation (DeVallance et al., 2019; Zhang et al., 2003). Overall, diabetes promotes adventitial fibroblast-driven inflammation and fibrosis in the vessel wall through phenotypic switching and enhanced paracrine activity.

#### 5.3.1 Signaling pathways in adventitial fibroblasts implicated in diabetes

In hyperglycemic conditions, adventitial fibroblasts secrete excessive extracellular matrix components. Increased glucose upregulates Discoidin Domain Receptor 2 (DDR2), a collagen receptor tyrosine kinase, which augments the transcription of fibronectin and collagen type I genes via stimulation of TGF-β1/SMAD2/3 signaling. Metformin can attenuate this response by inhibiting TGF-β1 activation and subsequent SMAD-mediated DDR2 expression, thus mitigating collagen overproduction and adventitial fibrosis (Titus et al., 2022). Our past work had identified DDR2 as a ‘master switch’ in cardiac fibroblasts, with an obligate regulatory role in their phenotypic transformation into α-SMA-expressing myofibroblasts, and in collagen type 1 expression, G1-S transition and apoptosis resistance (George et al., 2016; Titus et al., 2020; V et al., 2019). Our studies have also shown that hyperglycemia increases the expression of DDR2, collagen type I and fibronectin in isolated rat aortic adventitial fibroblasts and VSMCs via the TGF-β1/SMAD2/3 pathway, which is attenuated by resveratrol (Titus et al., 2022). Gene knockdown and overexpression indicated that hyperglycemia-induced increase in pro-fibrotic gene expression in these cells is mediated by DDR2 (Titus et al., 2022). Further, our study pointed to DDR2 as a molecular link between arterial fibrosis and diet-induced metabolic syndrome in a non-human primate model (Ushakumary et al., 2019).

Notably, Yu et al. (2021) demonstrated that DDR2 is highly expressed within the fatty and necrotic cores of human, rabbit, and mouse atherosclerotic plaques. They found that proatherosclerotic ox-LDL upregulates DDR2 expression in vascular smooth muscle cells (VSMCs) via the MAPK signaling pathway. In contrast, knocking down DDR2 using siRNA significantly inhibits the proliferation, migration, and collagen I-induced matrix metalloproteinase (MMP) expression in these cells. DDR2 deficiency suppresses both the protein expression and enzymatic activity of MMP-2 by reducing ERK1/2 phosphorylation (Yu et al., 2021).

### 5.4 Role of adventitial fibroblasts in vascular aneurysm

**Figure 3:**
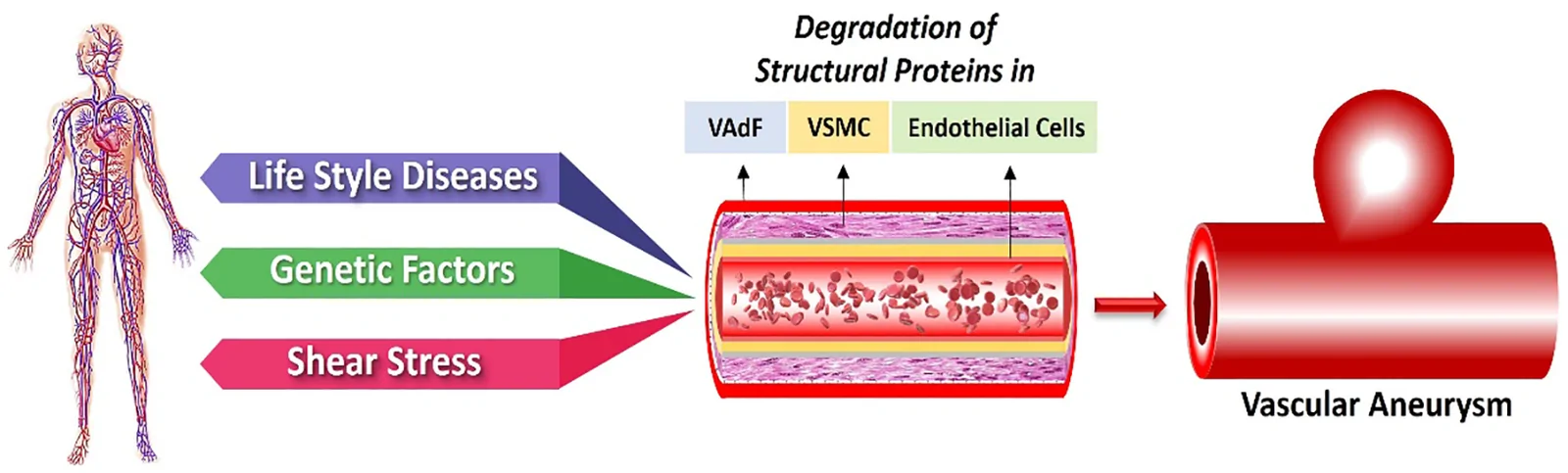
Key mechanisms involved in the development of aortic aneurysm.

Vascular aneurysm is an abnormal bulging of the vessel wall due to wall weakening following degradation of structural proteins, particularly collagen and elastin, which increases the risk of rupture (**Figure 3**). The most prevalent types of vascular aneurysms are Abdominal Aortic Aneurysm (AAA), Thoracic Aortic Aneurysm (TAA), Cerebral Aneurysm and Peripheral Aneurysms (Ardahanlı et al., 2025; Bozorgpour and Kim, 2025; Buja et al., 2025; Ganizada et al., 2024; Guo et al., 2024).

Development of aneurysm involves four important processes, including proteolysis, oxidative stress, inflammatory immune response and VSMC apoptosis. These processes can lead to loss of elasticity and resistance of the artery wall, compromising recovery of the normal arterial diameter after each pulsation of blood flow (Cho et al., 2023). The multifactorial nature of aortic aneurysm and its elusive pathophysiology limit available therapeutic interventions for preventing its progression (Mackay et al., 2022).

#### 5.4.1 Dysregulated ECM turnover

Endothelial damage, SMC loss and ECM degradation in the media and adventitia cause adverse remodeling of the aortic wall, leading to localized aortic aneurysm (Jana et al., 2019). Adventitial fibroblasts play a crucial role in aortic aneurysms and dissections via mechanisms involving inflammation, disruption of ECM and cell-cell interactions (Shen et al., 2020). It was demonstrated that Col1a1^+^ fibroblasts facilitate blood-brain barrier repair through the paracellular pathway in a TIMP metallopeptidase inhibitor 2 (TIMP2)-dependent manner, opening new avenues to treat intracerebral hemorrhage by targeting Col1α1^+^ fibroblasts and TIMP2 (Tsuruda et al., 2004). Varicella Zoster Virus (VZV)-infected human brain vascular adventitial fibroblasts exhibited enhanced gene expression pathways associated with vascular remodeling and vascular diseases (Bubak et al., 2022). A recent comprehensive review by Costa et al. (2023) dwells upon genetic, molecular and mechanotransductive factors underlying both syndromic and nonsyndromic arterial aneurysms, emphasizing how ECM imbalance and metalloproteinase overexpression weaken the arterial wall and predispose it to dilation and rupture (Costa et al., 2023).

#### 5.4.2 Collagen

The vasculature is enriched with type III collagen that maintains the structural integrity and tone of the vascular wall. Collagen staining reveals the presence of type III collagen in luminal and abluminal layers, but a complete deficiency of type III collagen associated with cerebral aneurysm is also reported (Neil-Dwyer et al., 1983; Ostergaard and Oxlund, 1987). While type VI collagen is normally observed exclusively in the adventitia, aneurysmal wall shows a different pattern in the abluminal and luminal layers. This changing pattern of type VI collagen in the context of aneurysms significantly modulates the ECM composition and organization in normal cerebral arteries (Mimata et al., 1997). Collagen in the vascular adventitial fibroblast layer plays a significant role in the pathogenesis of abdominal aortic AAAs and TAAs. Adventitial collagen fibers in AAA are less wavy, compared to normal (Urabe et al., 2016). Such microstructural changes in collagen fibril impact macromolecular ECM remodeling that determines cell-matrix interactions and the functional characteristics of aneurysm.

#### 5.4.3 Elastin

Dysregulated elastin turnover and remodeling processes may lead to the fragmentation, degradation or abnormal deposition of elastic fibers within the arterial wall, resulting in arterial stiffening and loss of vessel elasticity that mark aneurysm-prone vessels (Tai et al., 2016).

#### 5.4.4 Fibronectin, integrins and periostin

Dysregulation of fibronectin is implicated in the pathogenesis of aneurysms (Chen et al., 2023). Moreover, fibronectin interacts with MMPs, and MMP-2 and MMP-9 have gained attention for their role in promoting the initiation of intracranial aneurysms (Kim et al., 1997). Integrins in adventitial fibroblasts are also involved in force transmission from the ECM to the cytoskeleton (Chiquet et al., 2009), which can be an important factor in aneurysm formation. Further, in response to vessel wall stress, periostin can activate the expression of MMP-2 and MCP-1 that in turn can sustain aneurysm formation by triggering inflammation (Costa et al., 2023; Yamashita et al., 2013).

#### 5.4.5 Paracrine effects of vascular adventitial fibroblasts in aneurysm

Adventitial fibroblasts secrete factors that can promote inflammation, ECM remodeling and VSMC phenotypic switching that contribute to vascular remodeling and the pathogenesis of aneurysm (Mackay et al., 2022). They can also modulate endothelial cell phenotype and function, impacting vascular permeability, inflammation and angiogenesis within the aneurysm microenvironment.

##### 5.4.5.1 Vascular smooth muscle cells

Adventitial fibroblasts stimulate VSMC proliferation and migration, promoting intimal hyperplasia and aneurysm development. On the other hand, VSMCs exert paracrine effects on adventitia and protect the arterial wall against inflammation and proteolysis, and failure of this mechanism leads to aortic aneurysm (Kaneko et al., 2011). Using a mouse model of inducible fibroblast-specific loss of NADPH oxidase-2 (Nox2), a recent study demonstrated the critical function of fibroblast Nox2 in the development of Ang II-induced aortic vascular remodeling.

##### 5.4.5.2 Immune cells

In AAA, lymphocytes, macrophages and neutrophils populate the adventitial layer during aneurysm development. It is likely that macrophages drawn into the adventitia participate in the inflammatory process, triggering aneurysmal growth (Maiellaro and Taylor, 2007). A well-established mechanism in the development of aneurysms is adventitial inflammation, driven by invading macrophages (Dalal et al., 2025; Zhou et al., 2025). Recent studies showed that adventitial fibroblasts also play active, complementary roles in aneurysm pathobiology. In human AAA samples, high Fibroblast Activation Protein (FAP) expression, which is a marker of activated fibroblasts, correlated with increased macrophage accumulation in the aneurysm wall (Zhou et al., 2025). Further, platelet–fibroblast interactions show that fibroblasts can be induced to upregulate IL 6 and MMP 9 mediators that promote inflammation and matrix degradation in AAA models. Together, these findings indicate that, in addition to macrophages, adventitial fibroblasts actively drive ECM remodeling and local inflammation during aneurysm progression.

#### 5.4.6 Signaling pathways underlying ECM remodeling in vascular aneurysm

MMP-2, MMP-3, MMP-9, MMP-12, and MT1-MMP are consistently increased in aneurysmal adventitia. Their proteolytic activity leads not only to ECM loss but also to the activation of latent TGF-β and the continued activation of inflammation (Lin and Davis, 2023). Some matricellular proteins like WISP-1 (CCN4) and CYR61 (CCN1) are upregulated in aneurysmal adventitia. These secreted signaling proteins bind integrins and modulate proliferation, migration, ECM synthesis and inflammation. Deletion of WISP-1 reduces aneurysm severity and vascular wall cellular proliferation, suggesting its pathogenic relevance. In an Ang II–induced abdominal aortic aneurysm model of ApoE^-^/^-^ mice, genetic ablation of CCN4 led to significantly suppressed aneurysm progression despite equivalent blood pressure elevations compared to controls, and histological analysis revealed reduced cell proliferation in the vascular wall and attenuated adventitial remodeling (Schober et al., 2002; Williams et al., 2021). There are studies on integrin-mediated signaling and mechanotransduction (Xie et al., 2024). When fibroblasts sense altered ECM stiffness, integrin–focal adhesion kinase (FAK) complexes activate intracellular Rho/ROCK and YAP/TAZ pathways, promoting cytoskeletal tension, myofibroblast differentiation and collagen deposition. Tension-induced activation of MRTF-A further drives the expression of α-SMA and ECM genes, reinforcing pathological remodeling (Markella and Barbara, 2014). In aneurysm models, the balance between canonical and noncanonical TGF-β signaling is critical. ERK1/2 activation downstream of noncanonical TGF-β signaling leads to MMP production and vessel wall weakening, while Smad pathways support ECM integrity (Kubota et al., 2022).

### 5.5 Role of adventitial fibroblasts in aging

“You are as old as your arteries”- the famous statement by Thomas Sydenham, the English physician who rose to fame as English Hippocrates, suggests that vascular aging may reflect the damages to the arterial wall over the course of life (Weber and Mayer, 2020). Vascular aging, also referred to as “heart age” in the Framingham study, is a normal process of life in which the vasculature undergoes structural alterations, functional decline and increased susceptibility to various age-related vascular conditions (Rodgers et al., 2019). Vascular aging is considered a critical factor in the development and progression of cardiovascular diseases like atherosclerosis (Ma et al., 2024).

As age advances, vascular adventitial fibroblasts undergo phenotypic and functional changes that contribute to age-related alterations in vascular structure and function. Vascular adventitial fibroblasts in aged arteries exhibit altered mechano-transduction properties and response to mechanical stress. Accumulation of senescent cells within the adventitial layer contributes to vascular aging phenotypes, including arterial stiffening and impaired wound healing. Further, the adventitia is a site for local immune responses in aging and early atherosclerosis, promoting vascular smooth muscle cell invasion into the intima (Campbell et al., 2012). Recent research suggests that, as individuals age, T lymphocytes infiltrate the adventitia, enhancing inflammation (Gräbner et al., 2009). The plasticity of adventitial fibroblasts and their involvement in fibrosis, inflammation and angiogenesis make them a critical player in vascular aging (Sarad et al., 2024; Tillie et al., 2020).

#### 5.5.1 Dysregulated ECM turnover, vascular stiffening and arterial elasticity

With advancing age, all three layers of the artery are marked by increased collagen deposition (Díez, 2007; Fleenor et al., 2010, 2012, 2012; Wang et al., 2007; Wang and Lakatta, 2002). Adventitial fibroblast-mediated alterations in ECM turnover contribute to age-related vascular stiffening and reduced arterial elasticity. *In vitro* studies demonstrate that adventitial fibroblasts synthesize more collagen than SMCs (Patel et al., 2000) and the synthesis and deposition of collagens I and III by adventitial fibroblasts increase with age (Fleenor et al., 2010, 2012). It has been suggested that aging can induce adventitial TGF-β1-related oxidative stress, triggering the switch to the pro-synthetic myofibroblast phenotype associated with increased collagen deposition (Fleenor et al., 2010). Further, elastin is expressed by all cells within the artery, including adventitial fibroblasts, and aging causes decrease in elastin content in the arteries.

#### 5.5.2 Paracrine effects in the vessel wall during aging

A recent study revealed that the senescence-associated secretory phenotype (SASP) from endothelial cells exerts paracrine effects on the proteome of non-senescent adventitial fibroblasts and influences their metabolic and apoptotic pathways (Sarad et al., 2024). Moreover, dysregulated paracrine interactions between fibroblasts and other matrix-producing cells may disrupt tissue integrity and exacerbate age-related vascular pathologies such as arterial fibrosis and calcification. Adventitial cells respond to age-related changes in the microenvironment, and through paracrine signaling, interact with endothelial cells, VSMCs and immune cells to influence vascular aging. Paracrine interactions between adventitial fibroblasts and VSMCs are reported to modulate VSMC phenotype and function (Xie et al., 2025).

#### 5.5.3 Signaling pathways mediating ECM remodeling in aging

As noted above, one of the earliest changes that occurs in the aged adventitia is increased deposition of fibrillar collagens I and III, resulting in arterial stiffening. Adventitial fibroblasts are the main source of this collagen accumulation, stimulated by mechanotransduction signals from integrin-FAK complexes that transduce increased ECM stiffness (e.g., Rho/ROCK, YAP/TAZ, MRTF-A) to enhance collagen gene expression and myofibroblast differentiation. This stiffening loop mirrors changes in cardiac fibroblasts and aging vessels, where YAP/TAZ activation amplifies TGF-β–driven profibrotic responses (Mammoto et al., 2022). In addition, age-associated degradation of elastin fibers and reduced elastin to collagen ratio reduce adventitial elasticity (Xiao et al., 2023).

Further, with increasing stiffness, adventitial fibroblasts produce higher amounts of reactive oxygen species (ROS) via NADPH oxidases. ROS further promote inflammatory signaling (e.g., NF-κB) and activate latent TGF-β in the matrix, promoting fibrosis and oxidative stress. This oxidative and profibrotic milieu is compounded by ECM cross-linking through enzymes like lysyl oxidases (LOX/LOXL2/LOXL3) and non-enzymatic glycation, resulting in irreversible matrix stiffening (Dudek et al., 2023). Aging also elevates MMP levels, secreted by adventitial fibroblasts and inflammatory cells, leading to selective ECM degradation and the generation of bioactive matrix fragments. These fragments act as danger signals, activating pattern-recognition receptors and fueling NF-κB–mediated inflammation, which further disrupts ECM balance (Vidović and Ewald, 2022).

## 6 Sex-specific differences in fibroblast response

Significant sex-specific differences in fibroblast behavior have been observed in atherosclerosis. In pre-menopausal women, estrogen modulates inflammatory cytokine production and regulates fibroblast function by reducing excessive ECM turnover and matrix metalloproteinase activity (Poznyak et al., 2023). Adventitial fibroblasts from male mice were found to exhibit higher rate of collagen deposition and reduced expression of progenitor markers (e.g., Sca-1) with aging, which would impact wall stiffness (Gharraee et al., 2022). It is reported that sex hormones influence age-related increase in collagen deposition and reduce elastin integrity, resulting in a stiffer arterial wall (Sun, 2015). In females, estrogen helps maintain a balanced fibroblast phenotype, preserving ECM turnover and limiting excessive fibrosis during early aging. Post-menopausal loss of estrogen protection leads to comparable fibrotic responses in both sexes, ultimately increasing cardiovascular risk in older women (Lopez-Pier et al., 2018). It has been demonstrated that pulmonary artery adventitial fibroblasts (PAAFs) drive vascular remodeling in PAH through mechanisms heavily dictated by biological sex and ovarian hormones. Specifically, intact ovarian hormones act as a molecular regulator, raising the mechanical threshold required for female PAAFs to trigger profibrotic collagen synthesis in response to tissue stiffness. In contrast, male-derived (XY) and hormone-depleted female PAAFs exhibit heightened mechanosensitivity, causing them to aggressively deposit ECM (Lin et al., 2026).

## 7 Non-coding RNA-mediated regulation of vascular adventitial fibroblasts

Non-coding RNAs (ncRNAs) such as microRNAs (miRNAs) and long non-coding RNAs (lncRNAs) play a significant role in adventitial fibroblasts by controlling their proliferative, differentiative and ECM-producing behavior to maintain a functional vascular wall (Luo et al., 2023; Maegdefessel and Fasolo, 2024; Winter et al., 2023; Wu et al., 2018). Certain ncRNAs promote fibroblast proliferation and differentiation into myofibroblasts while others repress such activation and maintain vascular homeostasis (Wu et al., 2018). ncRNAs also regulate the synthesis and degradation of elastin and collagens, thereby impacting vascular structure and function (Luo et al., 2023, 2015; Ren et al., 2020; Song et al., 2020; Wang et al., 2014). Overexpressing miR-124 reduced fibroblast proliferation, migration and MCP-1 expression, while inhibiting miR-124 increased these activities in fibroblasts. The polypyrimidine tract–binding protein 1 is a direct target of miR-124, mediating its effects through the Notch1/PTEN/FOXO3/p21Cip1 and p27Kip1 signaling pathways (Cheung et al., 2006). It has been shown that miR-487b and miR-155 regulate the differentiation of adventitial fibroblasts into myofibroblasts (Wu et al., 2018). In hypertensive rats, a significant upregulation of miR-487b in the adventitia induces adventitial fibroblast differentiation (Wu et al., 2018). While over-expression of miR-29a-3p inhibits fibroblast activation, proliferation and ECM production, inhibition of miR-29a-3p mimics the effects of hypoxia.

miR-122-5p regulates apoptosis, oxidative stress and autophagy in aortic adventitial fibroblasts and its inhibition prevents apoptosis, oxidative stress and inflammation (Song et al., 2020). When over-expressed, miR-122-5p induces cellular damage through suppression of SIRT6 and ELA, suggesting that modulating the SIRT6-ELA-ACE2 pathway with miR-122-5p could be a novel therapeutic strategy to control vascular remodeling and dysfunction. Moreover, miR-122-5p may serve as a predictive biomarker for the detection of adventitial injury and vascular remodeling (Song et al., 2020). In rat aortic adventitial fibroblasts, miR-155 suppresses AT1R activity (Pacurari and Tchounwou, 2015). miR-155 overexpression in adventitial fibroblasts inhibited Ang II-induced ERK1/2 activation and also decreased α-SMA expression without affecting TGF-β1 levels, highlighting the role of miR-155 in adventitial fibroblast differentiation and the suppression of AT1R expression (Wang et al., 2014). Li et al., 2011 showed that severe hypoxia-induced pulmonary hypertension is characterized by the emergence of a distinct population of pulmonary artery adventitial fibroblasts that exhibit a constitutively active proinflammatory phenotype. These fibroblasts produce elevated levels of proinflammatory cytokines, chemokines and adhesion molecules that actively recruit, retain, and transition monocytes and macrophages into an activated proinflammatory and profibrogenic state. It is driven by epigenetic alterations, specifically evidenced by a significant increase in the expression and catalytic activity of class I histone deacetylases (HDACs). Applying small-molecule class I HDAC inhibitors markedly suppressed the production of these inflammatory mediators and diminished the ability of fibroblasts to induce monocyte migration and activation (Li et al., 2011).

**Figure 4:**
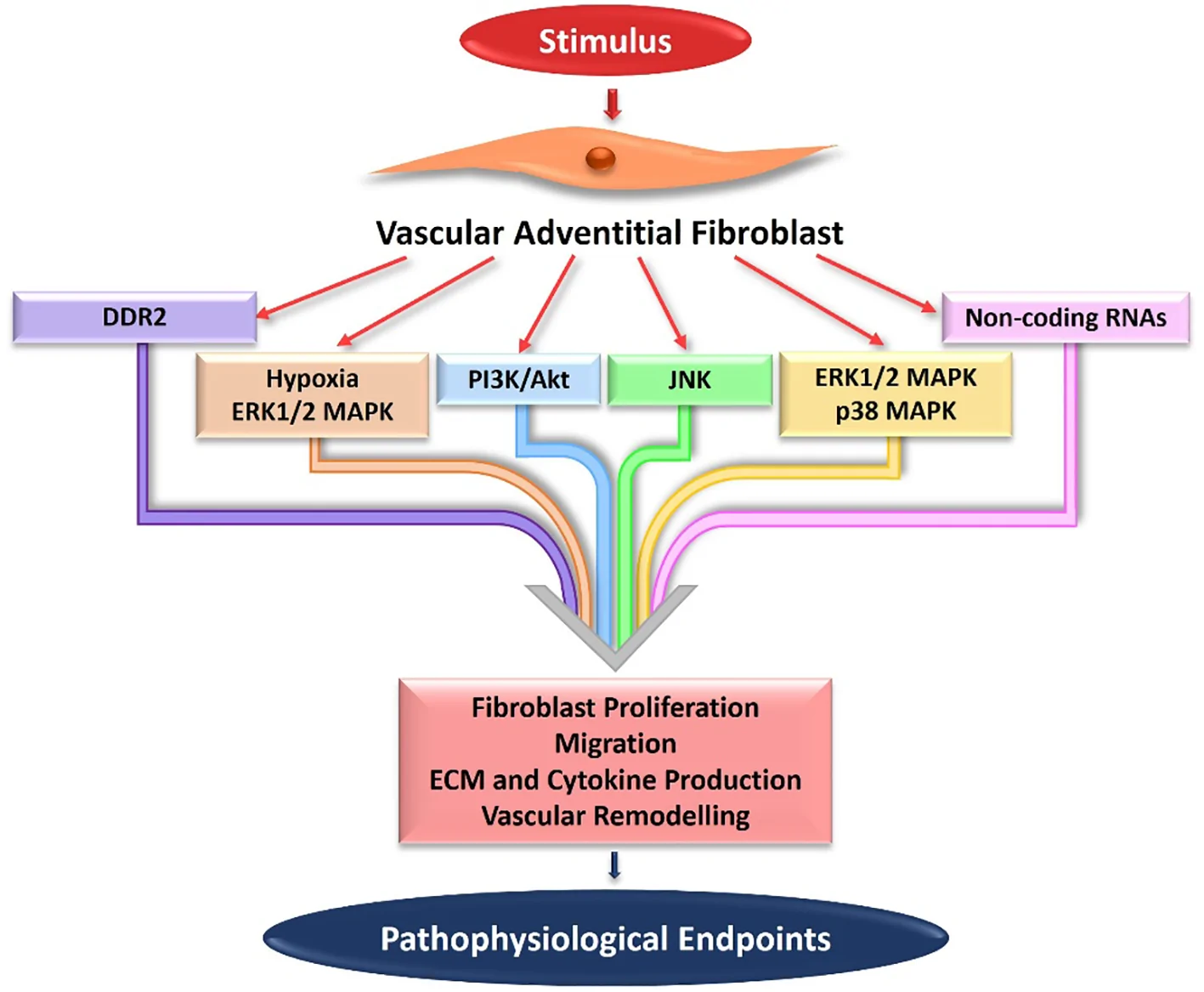
Key molecular mechanisms underlying adventitial fibroblast function.

It is amply clear from the preceding sections that multiple pathways underlie the response of adventitial fibroblasts in different pathophysiological contexts, contributing to the pathogenesis of vascular diseases (**Figure 4**).

## 8 Adventitial fibroblasts – the road ahead

**Figure 5:**
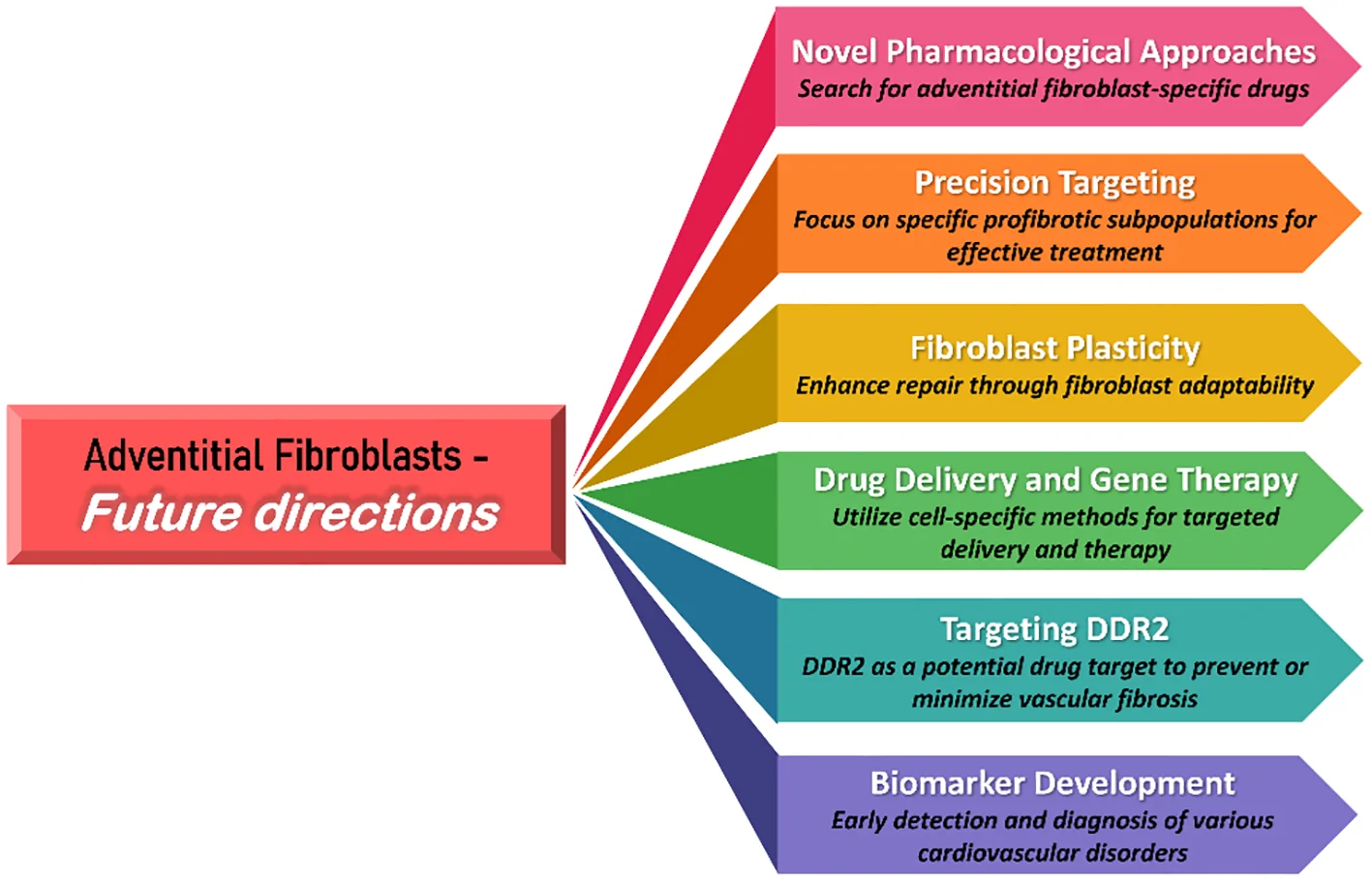
Emerging strategies and potential therapeutic options for understanding and modulating adventitial fibroblast function in vascular diseases.

Adventitial fibroblasts are important determinants of vascular health. Understandably, there is increasing interest in strategies that target this important cell type to achieve therapeutic benefits against a plethora of vascular conditions (**Figure 5**; **Table 3**).

**Table 3:** Emerging therapeutic strategies targeting vascular adventitial fibroblasts.

Therapeutic strategy	Mechanism of action	Potential benefits
Matrix Metalloproteinase (MMP) Inhibitors	Inhibit MMP activity to prevent ECM degradation and abnormal remodeling	Reduce vascular fibrosis and stiffness
Anti-inflammatory Agents	Suppress inflammatory cytokine production to modulate fibroblast activation	Decrease adventitial inflammation and subsequent fibrosis (Sarad et al., 2024)
Extracellular Vesicle-Based Therapies	Utilize vesicles to deliver regulatory molecules that influence fibroblast behavior	Promote healthy vascular remodeling and repair (Zhang and Sun, 2022)
ANGPTL4 Modulation	Target ANGPTL4 to influence fibroblast activity and ECM production	Potentially reduce fibrosis and improve vascular function (Zhu et al., 2023)
miRNA-based Therapy	Regulate fibroblast activation genes	Regulate fibroblast response (Guo et al., 2024)

### 8.1 Search for drugs that target adventitial fibroblasts

Fenofibrate, a PPARα agonist, inhibits apoptosis in vascular adventitial fibroblasts and upregulates SIRT1 expression in ApoE(-/-) mice (Wang et al., 2015). Fenofibrate also prevents TNF-α-induced apoptosis and cell cycle arrest in vascular adventitial fibroblasts *in vitro* (Wang et al., 2013). The PPARα-mediated effects of fenofibrate on apoptosis and SIRT1 expression involve the deacetylation of FoxO1 by SIRT1, a mechanism by which fenofibrate may protect against cardiovascular diseases by modulating adventitial fibroblast apoptosis through the SIRT1-FoxO1 pathway (Wang et al., 2013). The study also showed that the alkaloids, rhynchophylline, isorhynchophylline and rhynchophylla, may influence hypertension by promoting vascular adventitial fibroblast apoptosis and inhibiting their proliferation in the thoracic aorta of SHR (Dai et al., 2012). There is compelling rationale to develop drugs that selectively target adventitial fibroblasts to mitigate vascular pathologies.

### 8.2 Precision targeting of pro-fibrotic subpopulations

Since adventitial fibroblasts are not a uniform cell population but comprise multiple subtypes with unique molecular signatures and functional behavior, deciphering which subpopulations drive detrimental processes may help design targeted therapies to selectively modulate their activity. Recent advances in single-cell transcriptomic studies have identified fibroblast subsets with high pro-fibrotic potential that readily transition to myofibroblasts under stress conditions (Shi et al., 2021). Future therapies may utilize small molecules to specifically inhibit the signaling pathways (e.g., TGF-β, hedgehog or PDGF receptor pathways) that promote this transition. For example, blocking the TGF-β signaling cascade in the subset prone to myofibroblast conversion could reduce fibrotic ECM production while sparing fibroblasts involved in normal repair processes (Frangogiannis, 2020).

### 8.3 Fibroblast plasticity for repair

Future interventions may aim to reprogram activated fibroblasts back to a quiescent or regenerative state using epigenetic modulators, microRNA-based therapies or specific growth factor combinations. Such strategies would help restore normal vessel function and prevent progression to irreversible fibrosis (Anokye-Danso et al., 2012).

### 8.4 Cell-specific drug delivery and gene therapy

Recent advances in nanotechnology and genetic engineering offer promising approaches to target specific fibroblast subpopulations in the adventitia. For example, nanoparticles conjugated with antibodies against unique cell-surface markers (such as PDGFRα or Thy-1 variants expressed on pro-fibrotic fibroblasts) could deliver therapeutic payloads directly to these cells, minimizing off-target effects (Anokye-Danso et al., 2012). This is particularly important as recent single‐cell transcriptomic analyses have revealed extensive heterogeneity among vascular adventitial fibroblasts, identifying distinct profibrotic subpopulations with unique transcriptional programs. For example, van Kuijk et al. (2023) found three fibroblast trajectories marked by CD55, CXCL14 and LOX, each linked to different gene ontologies that expand in aged or hypercholesterolemic arteries (van Kuijk et al., 2023). Fries et al. (2025) identified four clusters of adventitial fibroblasts whose gene expression and proportions change during experimental atherosclerosis (Fries et al., 2025). Importantly, these studies have defined fibroblast‐specific markers (PDGFRA, DPEP1) in the arterial adventitia (van Kuijk et al., 2023), suggesting strategies for selective targeting that could minimize off‐target effects. Recent treatment strategies employ targeted delivery methods like engineered EVs or nanoparticles loaded with miRNAs or anti-fibrotic medications with ligands to target pathogenic fibroblasts, increasing local efficacy and lowering systemic toxicity (Zong et al., 2024). As a proof‐of‐concept, EVs engineered with integrin‐α5‐targeting peptides and loaded with the anti‐fibrotic miR‐138‐5p and pirfenidone were shown to selectively reprogram activated fibroblasts *in vivo* (Zhou et al., 2024).

### 8.5 DDR2 as a potential drug target to prevent or minimize vascular fibrosis

Robust evidence supporting the obligate role of DDR2 in collagen expression in cardiac fibroblasts (George et al., 2016) led to a special editorial that examined whether DDR2 is “the receptor we have been looking for” (DeLeon-Pennell, 2016). Further, given the indispensable role of DDR2 in pro-fibrotic gene expression in vascular adventitial fibroblasts and VSMCs (George et al., 2016; Titus et al., 2022; Ushakumary et al., 2019), targeting DDR2 in these cells seems a promising strategy to prevent excessive matrix deposition in the vessel wall and consequent vascular dysfunction. Moreover, the observation that anti-diabetic metformin and resveratrol may exert their vasoprotective, anti-fibrotic effects by inhibiting DDR2 (Titus et al., 2022; Ushakumary et al., 2019) lends credence to the postulation.

### 8.6 Biomarker development for early intervention

Understanding fibroblast heterogeneity would also facilitate the identification of cell-specific biomarkers that in turn would improve early detection of vascular remodeling and fibrotic progression. Adventitial fibroblast-targeted therapeutics leveraging advances in single-cell biology, genetic lineage tracing and targeted drug delivery hold immense promise because they combine precise molecular insights with innovative delivery systems.

## 9 Conclusion

Robert M Sapolsky remarked that “The purpose of science is not to cure us of our sense of mystery and wonder, but to constantly reinvent and reinvigorate it.” Recent advances in vascular biology have served to constantly reinvent and reinvigorate our sense of mystery and wonder over the intricate mechanisms that operate in harmony to ensure vascular health.

Be that as it may, this article offers an overview of vascular pathobiology and the key role played by vascular adventitial fibroblasts across various vascular conditions. The focus on atherosclerosis, hypertension, diabetes, aneurysm formation and aging uncovers the myriad roles of adventitial fibroblasts in response to distinct pathological stimuli. Apart from their critical role in the pathogenesis of atherosclerosis through orchestrating ECM remodeling, immune cell recruitment and plaque stability, adventitial fibroblasts exert paracrine effects and modulate the vascular microenvironment and disease progression. The pivotal role of adventitial fibroblasts in the pathogenesis of aneurysm, especially their contribution to ECM remodeling, inflammation and vascular remodeling, is of immense clinical importance. Unravelling the molecular mechanisms underlying fibroblast-mediated aneurysm development may eventually help prevent aneurysm progression and rupture. Several lines of evidence foreground the impact of adventitial fibroblasts on the aging vasculature. This article examines the role of adventitial fibroblasts in age-associated changes in matrix composition, cellular interactions and oxidative stress responses.

Looking ahead, the insights from the review may fuel the quest for an in-depth understanding of adventitial vascular fibroblast biology and its implications for vascular health and disease. In tandem with cutting-edge technologies such as single-cell sequencing, spatial transcriptomics and organ-on-a-chip models, researchers could dive deep into the heterogeneity and plasticity of adventitial fibroblasts across different vascular contexts. The identification of novel therapeutic targets, including microRNAs, ECM components and signaling pathways, holds promise for the development of precision medicine approaches tailor-made for personalized treatment.
